# Multi-target regulation of pro-inflammatory cytokine production by transcription factor Blimp-1

**DOI:** 10.1007/s00011-022-01671-2

**Published:** 2022-11-20

**Authors:** Qiushi Qin, Rui Li, Lan Li, Yue Zhang, Shuwei Deng, Liuluan Zhu

**Affiliations:** 1 Institute of Infectious Diseases, Peking University Ditan Teaching Hospital, Beijing, 100015 China; 2 Beijing Key Laboratory of Emerging Infectious Diseases, Institute of Infectious Diseases, Beijing Ditan Hospital, Capital Medical University, Beijing, 100015 China; 3 Beijing Institute of Infectious Diseases, Beijing, 100015 China; 4 National Center of Infectious Diseases, Beijing Ditan Hospital, Capital Medical University, Beijing, 100015 China

**Keywords:** Blimp-1, Cytokine storm, Macrophage, Toll-like receptor, NF-κB, Nuclear translocation

## Abstract

**Objective:**

Cytokine storm syndrome is a fatal condition related to infectious and autoimmune diseases. Here, we aim to investigate the regulatory mechanisms of Blimp-1 on multiple cytokine production.

**Methods:**

The *Blimp1* shRNA was transfected into RAW264.7 macrophages, followed by Toll-like receptor (TLR) ligand stimulation. The mRNA and protein levels of cytokines were detected by real-time PCR and flow cytometric bead array. The nuclear translocation of AP-1 and NF-κB p65 was measured by immunofluorescence staining. The transcriptional activity was detected by luciferase reporter assay with 5 × NF-κB reporter or with IL6 promoter reporter.

**Results:**

Blimp-1 significantly inhibited the expression and secretion of IL-1β, IL-6, and IL-18 in macrophages during stimulation with a variety of TLR ligands. The immunofluorescence staining results showed that Blimp-1 strictly controlled the nuclear translocation of NF-κB p65 in LPS-challenged macrophages. Furthermore, Blimp-1 directly inhibited the transcriptional activity of NF-κB and the transcription of IL6 gene.

**Conclusion:**

Blimp-1 represses the production of multiple pro-inflammatory cytokines by directly binding the genomic region and restricting the nuclear translocation and transcriptional activity of NF-κB. This finding may provide potential therapeutic strategies for the cytokine storm-related diseases.

**Supplementary Information:**

The online version contains supplementary material available at 10.1007/s00011-022-01671-2.

## Introduction

Cytokine storm syndrome is a fatal condition that occurs when the immune system responds aggressively in infectious diseases, autoimmune diseases, and CAR T-cell therapy. Cytokine storm plays a key regulatory role in sepsis, unfortunately, so far, most clinical trials simultaneously targeting multiple cytokine production or effect failed [1]. Drugs blocking TNF-α and IL-6 have been approved for the treatment of rheumatoid arthritis, but the blockade of a single cytokine has a limited therapeutic effect on the disease [2]. Therefore, therapies targeting multiple pro-inflammatory cytokines may provide new ideas for the treatment of cytokine storm-related diseases.

Transcription factor B lymphocyte-induced maturation protein (Blimp-1) is a key regulator of plasma cell differentiation in B cells and effector/memory function in T cells [3, 4]. Blimp-1 is also required to maintain immune tolerance in myeloid cells and embryonic intestine [5, 6]. For example, conditional deletion of Blimp-1 in dendritic cells (DCs) showed an increased production of IL-6 and resulted in aberrant activation of the adaptive immune system in systemic lupus erythematous [7]. As mentioned above, Blimp-1 acts as a “Brake pad” in regulating immune functions; however, the regulatory mechanism of Blimp-1 in myeloid cells remains unclear. Blimp-1 is a SET domain and zinc finger-containing transcriptional repressor. Our previous study has demonstrated that it directly binds to the promoters of PD-1 and TIGIT in T cells [8]. In the present study, we aim to reveal the potential roles and mechanisms of Blimp-1 in regulating multiple cytokine production in macrophages. The results may provide new insight into the mechanisms of cytokine storm formation and potential therapeutic targets.

## Materials and methods

See supplementary material.

## Results

**Fig. 1:**
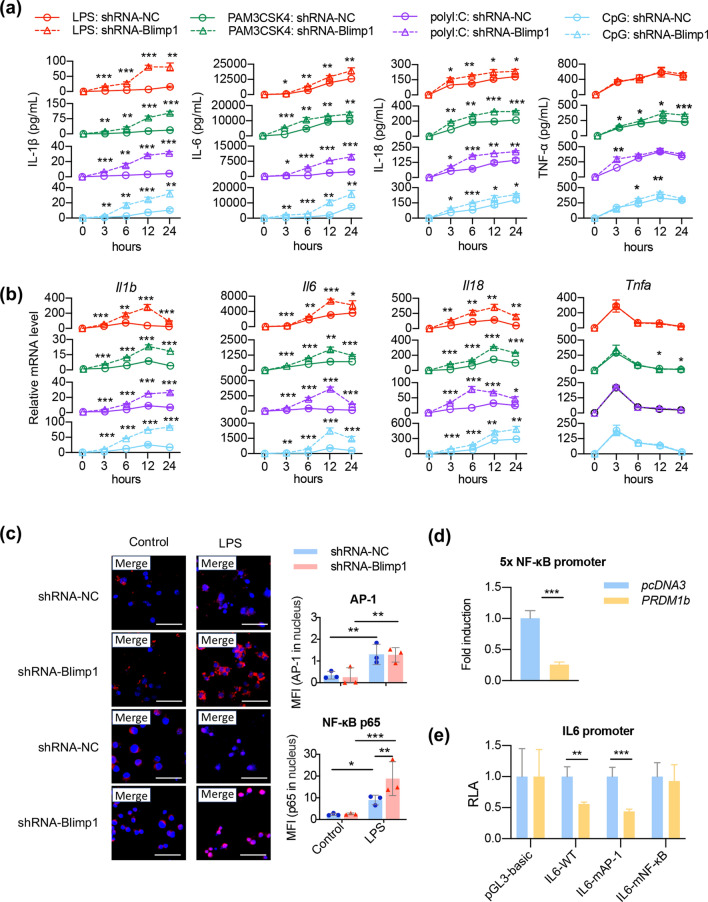
The effect of Blimp-1 on the production of pro-inflammatory cytokines and on the transcriptional activity of NF-κB. **a** The cells were transfected with *Blimp-1* shRNA or non-specific control and then stimulated with Pam3CSK4, PolyI:C, LPS, and CpG-ODN for 3, 6, 12 and 24 h. The contents of IL-1β, IL6, IL18, and TNF-α in the culture medium were detected by flow cytometric bead array analysis. **b** The mRNA levels of *Il1b*, *Il6*, *Il18*, and *Tnf* in RAW264.7 cells. **c** Immunofluorescence staining of AP-1 (red), NF-κB p65 (red), and DAPI (blue) in RAW 264.7 cells. The mean fluorescent intensity (MFI) of AP-1 and NF-κB p65 in nuclear was quantified and compared between groups. Scale bar = 20 μm. ***, *p* < 0.001; **, *p* < 0.01; *, *p* < 0.05. **d** Reporter gene assay was performed with 293 T cells transfected with a Gal4-p65 expressing plasmid and a reporter construct containing five copies of the NF-κB-binding site (5 × NF-κB), with either pcDNA3 or the Blimp-1 (PRDM1b) expressing plasmid. **e** Dual luciferase assay were performed with 293 T cells transfected with Blimp-1 (PRDM1b) expressing plasmid or the vehicle, as well as three reporters of IL6 promoter, wild type (WT), NF-κB-binding site mutation (mNF-κB), and AP-1-binding site mutation (mAP-1), respectively. RLA, relative luciferase activity

To elucidate the regulation of Blimp-1 on the pro-inflammatory cytokine production, we stably transfected RAW264.7 macrophages with *Blimp1* shRNA followed by stimulation with different TLR ligands to mimic invasive pathogens (Fig. S1). We stimulated the cells with Pam3CSK4 (TLR2 ligand), PolyI:C (TLR3 ligand), Lipopolysaccharide (LPS; TLR4 ligand), and CpG-ODN (TLR9 ligand) for 3, 6, 12, and 24 h, the secretion of cytokine IL-1β, IL-6, and IL-18 was significantly elevated by interfering with Blimp-1 (Fig. 1a). Consistently, by knocking-down *Blimp-1*, the mRNA levels of *Il1β*, *Il6*, and *Il18* were increased as early as 3 h after TLR ligand stimulations (Fig. 1b). Exceptionally, the expression and secretion of *Tnf* in *Blimp1* shRNA transfection was comparable to that in the non-specific control shRNA (Fig. 1a, b). By analyzing the Blimp-1 ChIP-seq data from a previous study [9], we observed that Blimp-1 had stable binding at both the proximal promoters of *Il1b* and *Tnf*, and the distal enhancers of *Il6* and *Il18* (Fig. S2). These results indicate that Blimp-1 has a strong inhibitory effect on a variety of pro-inflammatory cytokines at the transcription level during different infections.

TLRs activate the transcription factor NF-κB and activator protein-1 (AP-1), which consequently induces inflammatory responses. A hallmark activation of NF-κB and AP-1 is nuclear translocation, which is necessary for modulating gene transcription. By stimulating RAW264.7 cells with LPS, we found that Blimp-1 knockdown significantly up-regulated the expressions of p65 and AP-1 (Fig. 1c). Especially after *Blimp-1* was knocked out, it was remarkably increased in the cytoplasmic to nuclear translocation of p65 (Fig. 1c). These data suggest that Blimp-1 inhibits the transcription of multiple pro-inflammatory cytokines by restricting the entry of NF-κB p65 into the nucleus.

Moreover, we used a reporter construct containing five copies of the NF-κB-binding site (5 × NF-κB) to show an inhibitory effect of Blimp-1 on the transcriptional activity of NF-κB (Fig. 1d). We further constructed three reporters of IL6 promoter, wildtype (WT), NF-κB-binding site mutation (mNF-κB), and AP-1-binding site mutation (mAP-1), and performed dual luciferase reporter assays. We found that overexpression of Blimp-1 significantly inhibited the activities of WT and mAP-1 IL6 promoter; however, no distinct inhibitory effect was observed on the mNF-κB IL6 promoter (Fig. 1e). These data provide direct evidences that Blimp-1 controls the transcriptional activity and promoter binding of NF-κB.

## Discussion

Here, we demonstrated the inhibitory effect of Blimp-1 in pro-inflammatory cytokine production in macrophages. Blimp-1 has been reported to regulate myeloid cell terminal differentiation. Blimp-1 acts as a regulation of the macrophages pyroptosis in response to *L. donovani* infection [10]. Our findings further revealed the function and mechanism of Blimp-1 in maintaining appropriate inflammation in macrophages against infections. Since macrophages possess immunomodulatory and inflammatory abilities and actively participate in the development of many diseases, Blimp-1 may play a broad role in inflammatory diseases in which macrophages are involved.

We found that Blimp-1 directly binds to promoters or enhancers of IL-1β, IL-6, and IL-18 coding genes and inhibits nuclear translocation of NF-κB p65, thereby reducing the cytokine secretion. Notably, although Blimp-1 binds to the *Tnf* promoter, it does not inhibit the expression and secretion of TNF-α. There are two possible explanations. First, transcriptional repression by Blimp-1 involves recruitment of histone deacetylase, and the inability to regulate *Tnf* transcription may be due to dysregulation of histone deacetylation [11]. Second, there may be other transcription factors that act antagonistically to regulate *Tnf* transcription. Therefore, the transcriptional regulation mechanism of TNF-α still needs to be further elucidated.

In conclusion, Blimp-1 tightly controls the expression and secretion of multiple pro-inflammatory cytokines, which may be the key hub for regulating the inflammatory response. Although it is still necessary to further demonstrate the pathophysiological role of Blimp-1, this study provides new ideas and potential therapeutic strategies for the cytokine storm-related diseases.

## Supplementary Information

Below is the link to the electronic supplementary material.

Supplementary file1 (DOCX 34594 KB)

## Data Availability

All data generated or analyzed during this study are included in this article and its supplementary information file.
