# Supplementary material for: Multi-target regulation of pro-inflammatory cytokine production by transcription factor Blimp-1
Source: Inflamm Res. 2022 Nov 20;72(2):217–20. doi: 10.1007/s00011-022-01671-2 (PMC9925500; doi:10.1007/s00011-022-01671-2)
Supplement: Supplementary file 1 — Supplementary file1 (DOCX 34594 KB) [file 11_2022_1671_MOESM1_ESM.docx]

**Multi-target regulation of pro-inflammatory cytokine production by transcription factor Blimp-1**

Qiushi Qin^1,3#^, Rui Li^2,3,4#^, Lan Li^2,3,4^, Yue Zhang^2,3,4^, Shuwei Deng^2,3,4^, Liuluan Zhu^1,2,3,4^*

^1^ Institute of Infectious Diseases, Peking University Ditan Teaching Hospital, Beijing 100015, China

^2^ Beijing Key Laboratory of Emerging Infectious Diseases, Institute of Infectious Diseases, Beijing Ditan Hospital, Capital Medical University, Beijing 100015, China

^3^ Beijing Institute of Infectious Diseases, Beijing 100015, China

^4^ National Center of Infectious Diseases, Beijing Ditan Hospital, Capital Medical University, Beijing 100015, China

**Supplementary Materials**

**Methods**

**Cell culture and treatment**

The mouth peritoneal macrophage cell line RAW264.7 and the human renal epithelial cell line 293T were used. The RAW264.7 and 293T cell lines was cultured in Dulbecco's modified Eagles Medium (DMEM) (ThermoFisher Scientific, USA) containing 2 mM glutamine supplemented with 10% heat-inactivated fetal bovine serum (Gibco, USA) and 1% penicillin/streptomycin (Gibco, USA). Cells were incubated at 37 °C in a humidified atmosphere of 5% CO_2_. The RAW264.7 cells were seeded into 24-well plates and stimulated with 0.1 µg/ml lipopolysaccharide ([LPS] Sigma-Aldrich, # L4391), Pam3Cks4 (Sigma-Aldrich, # 506350), PolyI:C (Sigma-Aldrich, # P1530) and CpG-ODN 1668 (InvivoGen, # tlrl-1668) for 0, 3, 6, 12, 24 h at 37°C. The supernatant of RAW264.7 cells was tested by flow cytometric bead array analysis ([CBA] Biolegend, USA, #740847). The RAW264.7 cells were collected to extract total RNA by TRIzol reagent (Invitrogen, USA) according to the manufacturer’s protocol, these RNAs were then reverse-transcribed into cDNA by PrimeScript RT reagent Kit (TAKARA, Japan).

**The construction and stable transfection of shRNA**

The *Blimp1* shRNA sequences and a control shRNA were integrated into the lentiviral vector pWSLV-Sh08-GFP-Puro (Noweton Bioscience, China), and the constructed shRNA- pWSLV-Sh08-GFP-Puro plasmid was transfected into 293T cells. The supernatant was collected and filtered at 48 and 72 h after transfection. RAW264.7 cells were infected with shRNA lentiviral particles in the presence of 10 μg/ml PolyFect transfection reagent (Biowit Technologies, China). After two days of puromycin selection, RAW264.7 cells were collected for subsequent experiments. Primer sequences for *Blimp1* knockdown are presented in Table S1.

**Table S1. shRNA sequences used for Blimp1 gene knockdown**

| shRNA | Oligonucleotides (5’-3’) |
| --- | --- |
| ShRNA-*Blimp-1*-1 | AAAAGGTGCAGCCTTTATGAGTCCTCGAGGACTCATAAAGGCTGCACC |
| ShRNA-*Blimp-1*-2 | AAAACTCTCGACAGCAAATGGTTCTCGAGAACCATTTGCTGTCGAGAG |
| ShRNA-*Blimp-1*-3 | AAAAGCAGGATTACCCAAGAATACTCGAGTATTCTTGGGTAATCCTGC |

**Real-time qPCR analysis**

The *Il1b, Il6, Il18, Tnfa* and *Blimp1* gene was detected by Real-time qPCR analysis was performed using the TaqMan™ Universal PCR Master Mix (ThermoFisher Scientific, USA) according to the manufacturers’ instructions. Real-time qPCR was performed on 7500 Real-Time qPCR System (Applied Biosystems). The relative expression of mRNA was calculated by 2^-△△Ct^ method. The following primer pairs have been used: *Blimp1*_for 5-CTTCTCTTGGAAAAACGTGTGGG-3, *Blimp1*_rev 5- TCATATCAGCGTCCTCCATGT-3; *Il1b*_for 5-GAAATGCCACCTTTTGACAGTG, *Il1b* _rev 5-TGGATGCTCTCATCAGGACAG-3; *Il6*_for 5-CTGCAAGAGACTTCCATCCAG-3, *Il6*_rev 5-AGTGGTATAGACAGGTCTGTTGG-3; *Il18*_for 5- GTGAACCCCAGACCAGACTG-3, *Il18*_rev 5-CCTGGAACACGTTTCTGAAAGA-3; *Tnfa*_for 5- CAGGCGGTGCCTATGTCTC-3, *Tnfa*_rev 5- CGATCACCCCGAAGTTCAGTAG-3.

**Flow cytometric bead array analysis**

LEGENDplex™ Mouse M1 Macrophage Panel (8-plex) with V-bottom Plate (Biolegend, #740847) was used to detect the secretion levels of IL-1β, IL-6, IL-18 and TNFα in the supernatant of RAW264.7 cells stimulated by LPS (0.1 µg/ml), Pam3Cks4 (0.1 µg/ml), PolyI:C (0.1 µg/ml) and CpG-ODN 1668 (0.1 µg/ml). Every procedure followed the manufacturer's instructions.

**ChIP-Seq data analysis**

Blimp1 and input ChIP-Seq data downloaded from The Gene Expression Omnibus, accession number GSM1176410 and GSM1176408. Peak enrichment files are used to view the data in the IGV (Integrative Genomics Viewer 2.12.3) and all Blimp-1 binding site enrichment data are shown in the ChIP-Seq tracks.

**Immunofluorescence staining**

The antibodies against activator protein 1 (AP-1) (1:200 ABCAM, Cambridge, UK, #ab16911) and NF-kB p65 (1:300 ABCAM, Cambridge, UK, #ab64693) were used as primary antibodies, and Goat anti-rabbit IgG Alexa Fluor 555 (1:1000, ABCAM, Cambridge, UK, #ab150078) were applied as secondary antibodies. The nuclei were stained with Mounting Medium with DAPI-Aqueous, Fluoroshield (ABCAM, #ab104139). The samples were observed with Zeiss LSM 510 META confocal microscope. Images were performed by Image J.

**Transcriptional activity assay**

BLIMP1 (PRDM1b) cDNA was cloned into pcDNA3 plasmid as previous report [1]. The Gal4-p65 expressing plasmid and the five copies of the NF-κB binding site (5xNF-κB) reporter were kindly provided by Professor Tarik Möröy and described previously [2][3]. The wildtype IL-6 promoter sequence (-330bp/+5bp) were cloned into pGL3-basic luciferase reporter. The NF-κB and AP-1 binding site mutation including GGG (-60bp/-58bp) to TAC and CA (-265bp/-264bp) to TG respectively, as previously described [4]. The plasmids were transiently transfected into 293T cells as indicated using Lipofectamine 3000 (Thermo Fisher Scientific, Waltham, MA, USA) for 36 hrs and the luciferase activities were detected with Dual-Luciferase® Reporter Assay System (E1910, Promega, Madison, WI, USA).

## Statistics analysis

Unpaired t-test was used to compare shRNA-NC and shRNA-Blimp1 groups. Calculations were performed using the software package GraphPad Prism (GraphPad Software Inc., LaJolla, CA, USA). The graphical depiction of the *p* values within every experiment is displayed as follows: ***=p<0.001; **p<0.01; *=p<0.05.

**Reference**

[1] Zhu L, Kong Y, Zhang J, Claxton DF, Ehmann WC, Rybka WB, et al. Blimp-1 impairs T cell function via upregulation of TIGIT and PD-1 in patients with acute myeloid leukemia. J Hematol Oncol. 2017;10:124.

[2] Sharif-Askari E, Vassen L, Kosan C, Khandanpour C, Gaudreau MC, Heyd F, et al. Zinc finger protein Gfi1 controls the endotoxin-mediated Toll-like receptor inflammatory response by antagonizing NF-kappaB p65. Mol Cell Biol. 2010;30(16):3929-42.

[3] Zhu L, Meng Q, Liang S, Ma Y, Li R, Li G, et al. The transcription factor GFI1 negatively regulates NLRP3 inflammasome activation in macrophages. FEBS Lett. 2014 Nov 28;588(23):4513-9.

[4] Xiao W, Hodge DR, Wang L, Yang X, Zhang X, Farrar WL. Co-operative functions between nuclear factors NFkappaB and CCAT/enhancer-binding protein-beta (C/EBP-beta) regulate the IL-6 promoter in autocrine human prostate cancer cells. Prostate. 2004;61(4):354-370.

**
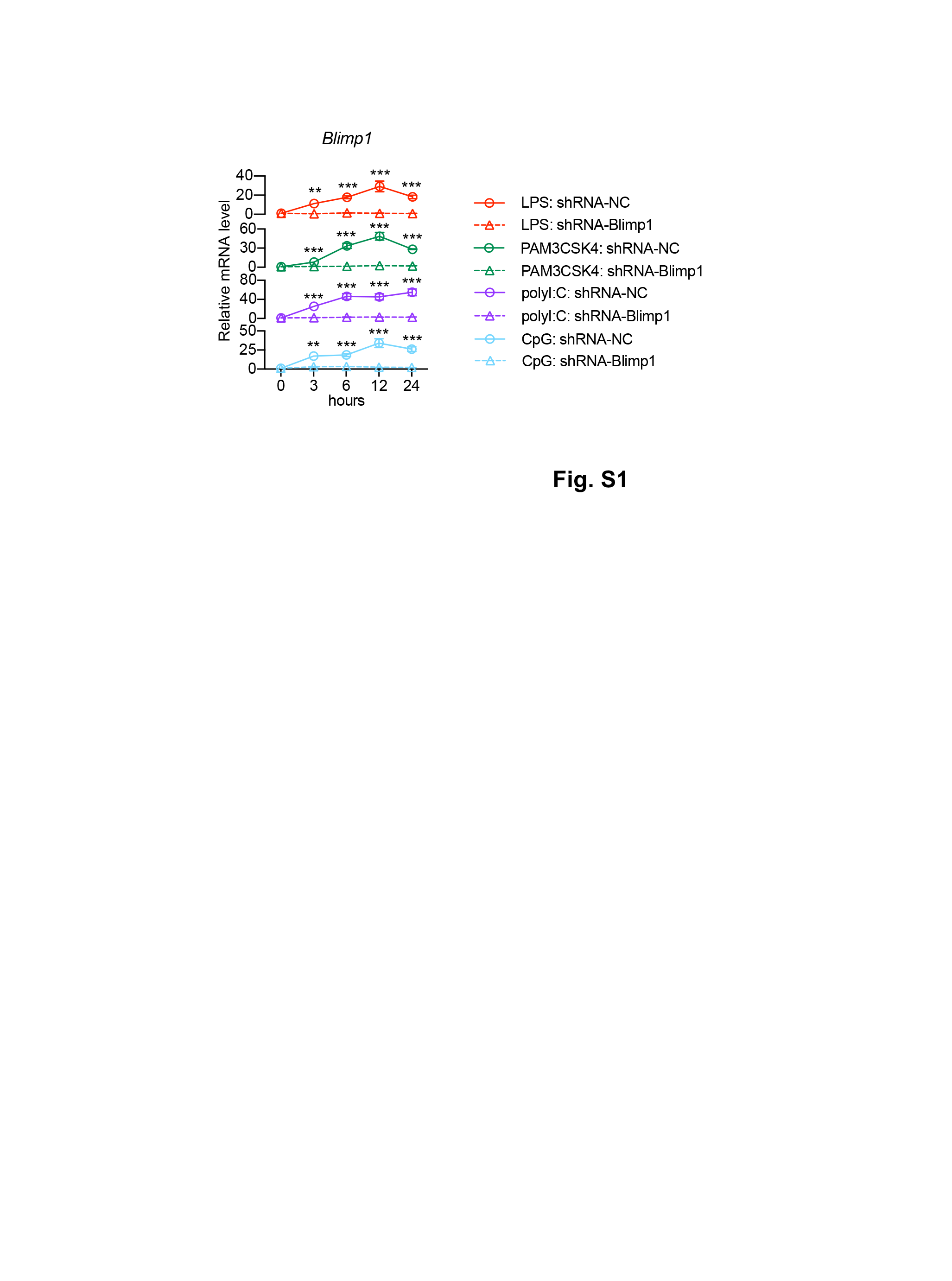

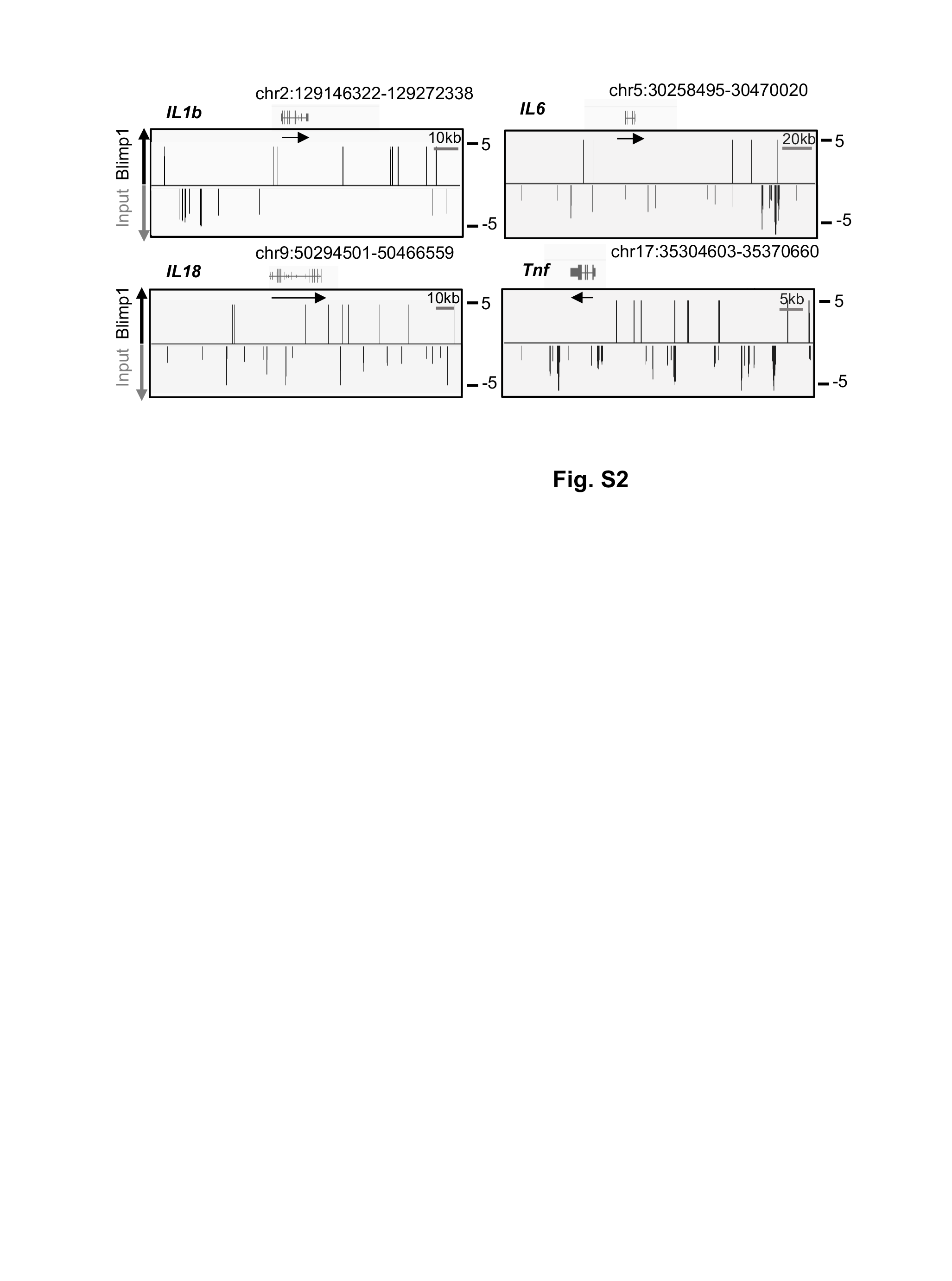
**

**Figure Legend**

**Fig S1. Effects of Blimp-1 on the production of pro-inflammatory cytokines in RAW264.7 cells. a** The mRNA levels of *Blimp-1* in RAW264.7 cells. The cells were transfected with *Blimp-1* shRNA or non-specific control and then stimulated with Pam3CSK4, PolyI:C, LPS, and CpG-ODN for 3, 6, 12 and 24 hours. ***, p<0.001; **, p<0.01; *, p<0.05.

**Fig S2. Identification of Blimp-1 binding sites with ChIP-seq.** The scale bars indicate the relative kbp scale on each gene, the arrow represents the transcript direction and the numbers on the right display the magnitude of sequence enrichment on a log2 scale. Peaks were identified with the IGV software and all Blimp-1 binding site enrichment data are shown in the ChIP-seq tracks.
